# Draft Genome Sequence of *Streptomyces* sp. Strain PRh5, a Novel Endophytic Actinomycete Isolated from Dongxiang Wild Rice Root

**DOI:** 10.1128/genomeA.00012-14

**Published:** 2014-04-17

**Authors:** Huilin Yang, Zhibin Zhang, Riming Yan, Ya Wang, Du Zhu

**Affiliations:** a Key Lab of Protection and Utilization of Subtropic Plant Resources of Jiangxi Province, Jiangxi Normal University, Nanchang, China; b Key Lab of Bioprocess Engineering of Jiangxi Province, Jiangxi Science and Technology Normal University, Nanchang, China

## Abstract

Here, we report the draft genome sequence of *Streptomyces* sp. strain PRh5 (China Center for Type Culture Collection [CCTCC] number 2013487), which is used to produce nigericin and nocardamine. The genome sequence will allow for the characterization of the molecular mechanisms underlying its beneficial properties.

## GENOME ANNOUNCEMENT

Nigericin is a polyether ionophore antibiotic derived from *Streptomyces hygroscopicus*. The structure was elucidated by X-ray crystallography in 1968 (1). Nigericin acts as an H^+^/K^+^/Pb^2+^ ionophore to chelate metal ions and transport them across cell membranes. In the past, nigericin was used as an antibiotic active against Gram-positive bacteria (2, 3). Recently, studies have shown that nigericin inhibits the Golgi functions in eukaryotic cells and exhibits anti-HIV activity (4, 5). Nocardamine, a hydroxamate siderophore, was initially isolated as an antibacterial metabolite of a *Nocardia* strain. Nocardamine exhibits antibacterial activity against mycobacterium species, especially tetracycline-resistant strains (6). *Streptomyces* sp. strain PRh5, which can produce nigericin and nocardamine, is a novel endophytic actinomycete that we isolated from Dongxiang wild rice root in China. *Streptomyces* sp. PRh5 was collected in the China Center for Type Culture Collection (CCTCC) with the collection number of CCTCC 2013487. Here, we report the first genome sequence of *Streptomyces* sp. PRh5, which we determined in an attempt to identify the nigericin and nocardamine biosynthetic gene clusters. The genome information may afford a basis for subsequent research directly connected with the natural product synthesis field.

The genome was sequenced using the Illumina Solexa HiSeq2000 instrument at the Beijing Genomics Institute (BGI) (Shenzhen, China). A library containing 500-bp inserts was constructed. Sequencing was performed with the paired-end strategy of (90, 90)-bp reads to produce 1.0 Gb of filtered sequences, representing 90-fold coverage of the genome. The sequences were assembled into 290 contigs using Velvet software (7).

Genome annotation was performed with the NCBI Prokaryotic Genome Annotation Pipeline 2.0. Open reading frames (ORF) were identified by Glimmer 3.02 (8) and Genemark (9). The resulting translations were used for a BLASTP (10) search against the GenBank NR database, as well as the KEGG (11) and COG (12) databases. tRNA and rRNA genes were identified by tRNAscan-SE (13) and RNAmmer (14), respectively.

The PRh5 chromosome is about 11.1 Mbp in length, with an average G+C content of 71.1%. A total of 8,712 protein-coding genes were identified. The genome sequence represents a valuable shortcut for helping scientists find genes. Putative nigericin and nocardamine biosynthetic gene clusters are found in the *Streptomyces* sp. PRh5 genome. Gene expression analysis and bioassays are needed for further investigation into these genes. The genome sequence will accelerate the progress of research involving *Streptomyces* sp. PRh5.

### Nucleotide sequence accession numbers.

This whole-genome shotgun project has been deposited at DDBJ/EMBL/GenBank under the accession number JABQ00000000. The version described in this paper is version JABQ01000000.
